# Detecting Unfavorable Driving States in Electroencephalography Based on a PCA Sample Entropy Feature and Multiple Classification Algorithms

**DOI:** 10.3390/e22111248

**Published:** 2020-11-03

**Authors:** Tao Zhang, Hong Wang, Jichi Chen, Enqiu He

**Affiliations:** 1 Department of Mechanical Engineering and Automation, Northeastern University, Shenyang 110819, China; 1310072@stumail.neu.edu.cn (T.Z.); jcchen@stumail.neu.edu.cn (J.C.); 2 College of Applied Technology, Shenyang University, Shenyang 110044, China; 3 School of Mechanical Engineering, Shenyang University of Technology, Shenyang 110870, China

**Keywords:** electroencephalography, entropy, unfavorable driving states, feature selection, classification

## Abstract

Unfavorable driving states can cause a large number of vehicle crashes and are significant factors in leading to traffic accidents. Hence, the aim of this research is to design a robust system to detect unfavorable driving states based on sample entropy feature analysis and multiple classification algorithms. Multi-channel Electroencephalography (EEG) signals are recorded from 16 participants while performing two types of driving tasks. For the purpose of selecting optimal feature sets for classification, principal component analysis (PCA) is adopted for reducing dimensionality of feature sets. Multiple classification algorithms, namely, K nearest neighbor (KNN), decision tree (DT), support vector machine (SVM) and logistic regression (LR) are employed to improve the accuracy of unfavorable driving state detection. We use 10-fold cross-validation to assess the performance of the proposed systems. It is found that the proposed detection system, based on PCA features and the cubic SVM classification algorithm, shows robustness as it obtains the highest accuracy of 97.81%, sensitivity of 96.93%, specificity of 98.73% and precision of 98.75%. Experimental results show that the system we designed can effectively monitor unfavorable driving states.

## 1. Introduction

Severe traffic accidents caused by unfavorable driving states, e.g., fatigue or drowsiness, are increasing rapidly with each passing year [1,2,3,4,5,6,7]. In addition to this, unfavorable driving states such as bad weather and insufficient light also cause quite a lot of vehicle crashes and are significant causes of traffic accidents [8,9]. Effective improvement of traffic safety and reduction of casualties and economic losses caused by traffic accidents due to unfavorable driving states are important tasks and are the mission of traffic management researchers around the world [10,11]. Therefore, it is very important and urgent to develop a system for detecting unfavorable driving states.

Relying on physiological signals to detect the driver’s state is considered to be an effective method and is widely used in related fields [12,13,14,15]. EEG signals, in particular, are the most predictive indicators for measuring brain electrical activity and can be integrated into the intelligent monitoring system of vehicles to better monitor driving states [16]. Researchers have attempted various computational approaches based on EEG signals for detection of unfavorable driving states. Fu et al. [16] proposed a driver fatigue detection system based on electroencephalogram signals using a relative operating characteristic curve (ROC) and gray relational analysis (GRA). In another study [12], the authors combined two novel functional brain network (FBN) approaches, synchronization likelihood (SL), minimum spanning tree (MST) and KNN classifier for recognition of driver drowsiness. Wali et al. [17] classified driver distraction level by using fusion of discrete wavelet packet transform and fast Fourier transform based on wireless EEG signals, and the highest classification accuracy they achieved was 85%. In another study [18], the authors proposed EEG alpha spindle measures based on fast Fourier transform (FFT) for driver fatigue detection. In [19], the authors proposed an alertness level detection based on the spectral power index of Pz–Oz sites through normalized Haar discrete wavelet packet transform.

Although the methods described above for detecting driver states have made remarkable progress, there is still room for improvement in the accuracy and robustness of detection systems based on EEG signals. An entropy-based method for measuring non-linear dynamic parameters of incidence of new information in a time series has been applied in many scientific fields such as assisted diagnosis of diseases [20], cognitive ability understanding [21], neuroimaging research [22] and quantification of brain function [23]. Inspired by this, we consider the EEG signal, especially in an actual driving environment, to have the characteristics of complexity, instability and non-linearity. We use an entropy-based method in this research as the feature extraction strategy of the EEG signal. The aim of this research is to design a robust system to detect unfavorable driving states based on analysis of EEG signals. Therefore, a new system combining sample entropy (*SampEn*) feature analysis [24] and multiple classification algorithms is proposed. In fact, *SampEn* evaluates the complexity of time series by assessing the possibility of generating new patterns in the signal. It is expected that: (1) the performance evaluation of multiple classification algorithms could prove that the feature extraction method based on sample entropy is more reliable and effective in recognizing the driver’s unfavorable state. (2) The proposed system combined with principal component analysis (PCA) and multiple classification algorithms could improve the ability to detect the driver’s state.

The organization of this paper is as follows: Section 2 introduces the materials and methodology, including experimental setup, data collection and preprocessing, feature extraction and classification algorithms. The results and discussions of experiment are shown in Section 3, which also contains a comparison of the performance of the proposed method with some published similar work. Finally, the conclusions of this research are derived in Section 4.

## 2. Materials and Methods

### 2.1. Experimental Setup

**Figure 1:**
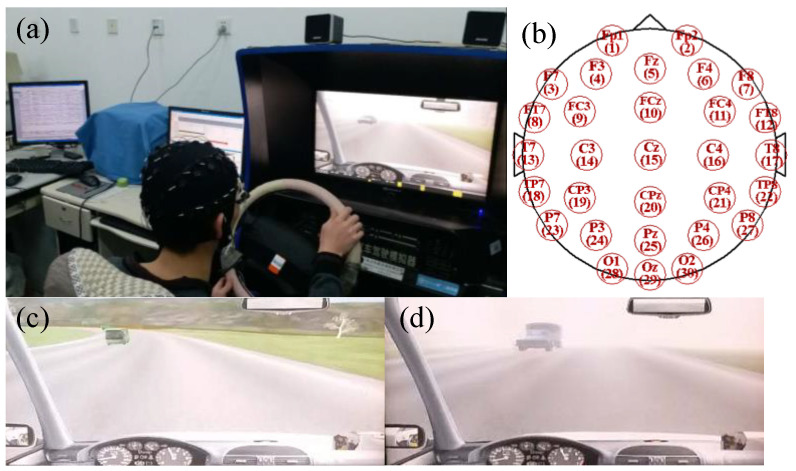
Experimental setup (**a**), electrodes position according to the International 10–20 system (**b**), Task A (**c**) and Task B (**d**).

**Figure 2:**
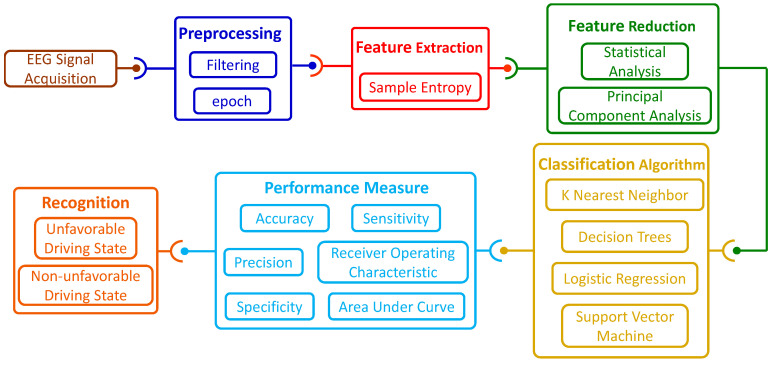
General block diagram of the proposed system.

The experiment was conducted in a temperature-controlled and sound-controlled room, using a driving simulator (see Figure 1a) produced by Beijing Jinggongkeye Scientific Education Co., Ltd. (Beijing, China). The driving simulator was made up of a steering wheel, a horn, a clutch, a brake pedal, an accelerator, a shift, a chair, a liquid crystal display (LCD) and turn signals. In this study, a total of sixteen male participants volunteered for the experiment. All of the participants were postgraduate students at the School of Mechanical Engineering and Automation, Northeastern University (NEU). The age of the participants ranged from 24 to 28 years (mean = 25.9, SD = 1.5 years). The experiments were approved by the local ethical committee of the Institutional Review Board, NEU. According to their self-reports, none of the participants had any history of psychological and neurological illness. All of them reported normal hearing and normal or corrected-to-normal vision. All participants owned C1 (manual transmission, MT) driving licenses. They were asked to sleep adequately before the experiment and to avoid drinking coffee, alcohol or tea during the experiment. They provided written consent prior to entering the experiment and received 50 Yuan (RMB) for participation in the experiment. In order to enable each participant to be proficient in using the driving simulator, each participant was required to continuously use the driving simulator for 20 min and was also required to wear an EEG acquisition cap during driving. While driving, the participants were prohibited from drinking, eating, listening to the radio, talking to others or adjusting the seats. After that, the participants were asked to perform two driving tasks, namely Task A and Task B, as shown in Figure 1c,d, respectively. Task A referred to the participants driving on a two-lane and 10-m wide rural road, and the vehicle speed had to be controlled below 50 km per hour. The road was flat and the traffic density was moderate. When the vehicle encountered incoming cars in the opposite lane for 50 times, Task A ended, and it took about 10 min. When performing Task A, the participants were relaxed during the driving process, which was also determined by the instructor’s inquiry after performing the task. For Task B, participants drove in the same driving environment as Task A (except for the weather conditions). Task B was driving in foggy weather, with visibility of about 200 m. Decreased visibility and reduced field of vision will increase driving difficulty and induce participants to drive in an unfavorable state. Especially when a car came in the opposite lane in foggy weather, the impact on the driver’s control of the vehicle was more serious, which also prevented them from making correct judgments on the trajectory of the vehicle in a shorter time. The situation was also confirmed by the instructor. Therefore, when a driver drove a vehicle in a foggy day and a vehicle was approaching in the opposite direction, the driver’s state at this time was considered to be an unfavorable driving state in this study. There was a 10-min interval between Task A and Task B for the participants to rest. Therefore, the time for each participant to complete the entire experiment was about 1 h. The general block diagram of the proposed system is shown in Figure 2.

### 2.2. Data Collection and Preprocessing

During the driving experiment, the EEG signals of each participant were captured in real time through a 40-channel Neuroscan EEG acquisition device. Two electrodes attached above and below the arch of the left eyebrow were used to record horizontal and vertical electrooculograms (EOG). All EEG channels were referred to another two electrodes placed on the left and right mastoids in accordance with the International 10–20 system of electrode placement [12], and electrode distribution is shown in Figure 1b. Electrode impedance was calibrated to be <5 kΩ and EEG signals were sampled at a rate of 1000 Hz. A time-stamp was marked for each piece of information that the vehicle met with incoming cars in the opposite lane by using STIM2 software. Contaminated EEG signals due to large drifts were removed by scanning. Additionally, preprocessing of data was carried out in SCAN 4.3 software. First, the EEG signal was previewed to remove obvious drifting fluctuations, the reject block was set at the start and end positions, and then the ocular artifact reduction was executed (min select, 20 ms, and duration set, 400 ms). Subsequently, baseline correction was performed to eliminate deviation of the EEG signal from the baseline. If the recorded EEG data was poor in stability or there was a serious baseline drift, we considered removing the data. Next, an artifact rejection operation was conducted to ensure that the reception accounted for more than 80% of the total. Maximum and minimum values were set to ±50 μV. Finally, the band pass frequency was set from 0.01 to 70 Hz (zero phase shift, finite impulse response (FIR) filter 24 dB/oct). The EEG signal from 30 channels was sectioned into 1-s epochs starting at −100 ms prior to onset of the marked time-stamp and ending at 900 ms, resulting in 50 epochs for each participant. With the 16 participants, a total of 800 epochs of the dataset were formed for unfavorable driving states and another 800 epochs for non-unfavorable driving states.

### 2.3. Feature Extraction

Sample entropy (*SampEn*) assesses the complexity of a time series by evaluating the probability of generating a new pattern in the signal. The concept of sample entropy was first proposed by Richman and Moorman [25] and has been applied in assessing the complexity of physiological time series and diagnosing pathological conditions [20,24,26]. Sample entropy is an improvement on approximate entropy, which has two advantages: the calculation of sample entropy does not depend on the length of the data and the sample entropy has better consistency, that is, the changes of the parameters *m* and *r* have the same degree of influence on the sample entropy. The algorithm is expressed as follows:

(1) For a given N-dimensional time series *u*(1), *u*(2), …, *u*(*N*), *u*(1), *u*(2), …, *u*(*N*).
(2) Reconstruct m-dimensional vector *X*(1), *X*(2), …, *X*(*N* − *m* + 1), where *X*(*i*) = [*u*(*i*), *u*(*i* + 1), …, *u*(*i* + *m* − 1)].
(3) For 1 ≤ *i* ≤ *N* − *m* + 1, count the number of vectors that meet the following conditions:
  (1) $$B_{i}^{m}(r)=\left(number\;of\;X(j)\;such\;that\;d[X(i),X(j)]\leq r\right)/\left(N-m\right),i\neq j$$
  where *d* is the distance between the vectors *X*(*i*) and *X*(*j*), which is determined by the maximum difference between the corresponding elements, and *r* is a real number, which represents the measure of ‘similarity’. The value range of j is [1, *N* − *m* + 1], but *j* ≠ *i*.
(4) Find the average of Formula (1):
  (2) $$B_{}^{m}(r)=(N-m+1{)}^{-1}\sum_{i}^{N-m+1}B_{i}^{m}(r)$$
(5) Repeat steps 3 and 4; *A^k^*(*r*) is obtained.
(6) The sample entropy (*SampEn*) is defined as:
  (3) $$SampEn=-\ln\left[A^{k}(r)/B^{m}(r)\right]$$
  Thus, lower values of *SampEn* imply a greater self-similarity of the time series; larger values of the *SampEn* imply more complex time series.

### 2.4. Feature Selection and Reduction

To search for the optimal feature sets, an automatic feature selection from the original feature sets is completed by principal component analysis (PCA). The main idea of PCA is to map n-dimensional features onto the k-dimension. This k-dimension is a brand-new orthogonal feature, also called the principal component, and it is a k-dimension feature reconstructed from the original n-dimension feature.

The maximum projection variance method is used in this research, which maximizes the variance of the data in the projected space. Furthermore, the direction with the largest data variance is selected for projection to maximize the difference of the data, so more original data information is retained.

### 2.5. Classification

In this research, various classification algorithms, namely, k nearest neighbor (KNN), decision tree (DT), support vector machine (SVM) and logistic regression (LR) have been studied and compared, as briefly introduced below. These classification algorithms are executed on MATLAB R2018b.

#### 2.5.1. K Nearest Neighbor

The k nearest neighbor (KNN) [27] classification algorithm is one of the simplest methods in data mining classification technology. K nearest neighbor refers to its k nearest neighbors, that is, each sample can be represented by its nearest k neighbors. The core concept of the KNN algorithm is that if most of the k nearest neighbors of a sample in the feature space belong to a certain category, the sample also belongs to this category and has the characteristics of the samples in this category. In this research, k values between 2 and 10 are considered, and distance metric and distance weights are set to Euclidean and squared inverse, respectively.

#### 2.5.2. Decision Tree

Decision tree (DT) is the process of classifying data through a series of rules [28]. Decision tree is also the most frequently used data mining algorithm. Intuitively, the decision tree classifier is like a flow chart composed of a judgment block and a termination block. The termination block represents the classification result (that is, the leaves of the tree). The judgment module represents the judgment on the value of a feature. The generation process of a decision tree is mainly divided into feature selection, decision tree generation and pruning. In this research, the maximum number of splits is set to 20, and split criterion is Gini’s diversity index.

#### 2.5.3. Support Vector Machine

Support vector machines (SVM) [11,29] show many unique advantages in solving small sample, non-linear and high-dimensional pattern recognition. They map the vector to a higher-dimensional space, in which a hyperplane with maximum separation is established. Two hyperplanes parallel to each other are built on both sides of the hyperplane that separates the data. A separating hyperplane is established with the proper direction to maximize the distance between two hyperplanes parallel to it. The assumption is that the greater the distance or spacing between parallel hyperplanes, the smaller the total error of the classifier. In this research, we utilize linear SVM structure, quadratic SVM structure and cubic SVM structure to classify the EEG signals.

#### 2.5.4. Logistic Regression

Logistic regression (LR) [30] is a classification algorithm. It is commonly used in binary classification problems. It converts input values into predicted values in linear regression, and then maps them to the sigmoid function. The values are used as the *x*-axis variable and the *y*-axis as a probability. The y value corresponding to the predicted value is closer to 1, indicating that it is fully in line with the predicted result. Compared with other classification algorithms, the mathematical model and solution of logistic regression are relatively simple and easy to implement.

### 2.6. Performance Measure

The performances of these four classifiers considered in this research are evaluated: sensitivity (SEN), specificity (SPE), precision (PRE) and accuracy (ACC). True positive (TP) is the number of unfavorable driving states classified as unfavorable driving states, and true negative (TN) is the number of non-unfavorable driving states identified as non-unfavorable driving states. False positive (FP) is the number of non-unfavorable driving states classified as unfavorable driving states, and false negative (FN) is the number of unfavorable driving states identified as non-unfavorable driving states. The equations of the performance measure parameters are as follows:

(4) $$\mathrm{SEN}=\frac{\mathrm{TP}}{\mathrm{TP}+\mathrm{FN}}\times 100\%$$

(5) $$\mathrm{SPE}=\frac{\mathrm{TP}}{\mathrm{TN}+\mathrm{FP}}\times 100\%$$

(6) $$\mathrm{PRE}=\frac{\mathrm{TP}}{\mathrm{TP}+\mathrm{FP}}\times 100\%$$

(7) $$\mathrm{ACC}=\frac{\mathrm{TP}+\mathrm{TN}}{\mathrm{TP}+\mathrm{TN}+\mathrm{FP}+\mathrm{FN}}\times 100\%$$

Usually, a receiver operating characteristic (ROC) graph is used to illustrate the relationship between two important quantities: true positive rate (TPR) and false positive rate (FPR) in an intuitive way. Moreover, the area under curve (AUC) of the ROC curve is between 0.1 and 1, which can be used as an intuitive value to evaluate the classifiers considered in this research. Precisely, the larger AUC value implies better performance of a classifier. Additionally, 10-fold cross-validation is used in this research to assess the performance of the classification algorithms considered.

## 3. Results

**Figure 3:**
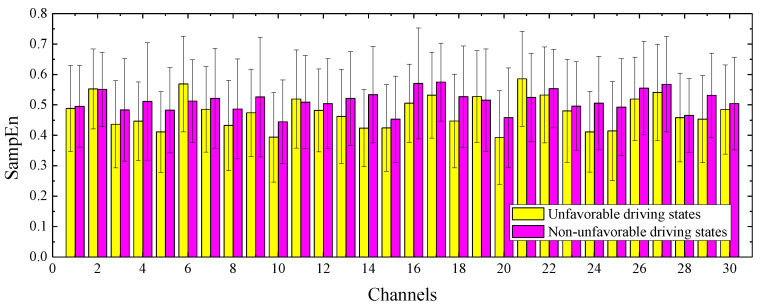
Mean and standard deviation of *SampEn* for each channel.

In this research, we computed *SampEn* for each channel of the preprocessed EEG signal. Figure 3 represents the mean and standard deviation of *SampEn* in each of the 800 unfavorable driving states and 800 non-unfavorable driving states of EEG signals from 16 participants. The *x*-axis and *y*-axis correspond to channels and sample entropy values, respectively, where the numbers on the *x*-axis and the lead names are as follows: FP1 (1), FP2 (2), F7 (3), F3 (4), FZ (5), F4 (6), F8 (7), FT7 (8), FC3 (9), FCZ (10), FC4 (11), FT8 (12), T7 (13), C3 (14), CZ (15), C4 (16), T8 (17), TP7 (18), CP3 (19), CPZ (20), CP4 (21), TP8 (22), P7 (23), P3 (24), PZ (25), P4 (26), P8 (27), O1 (28), OZ (29) and O2 (30). As seen in Figure 2, except for the four channels (F4, FC4, CP3 and FZ), the *SampEn* values of the other 26 channels of unfavorable driving states are generally lower than the non-unfavorable driving states. Table 1 also exhibits the results of Mann–Whitney U-tests performed on the *SampEn* feature (unfavorable driving states vs. non-unfavorable driving states) for each channel, and the *p* values less than 0.05 are marked in bold.

**Figure 4:**
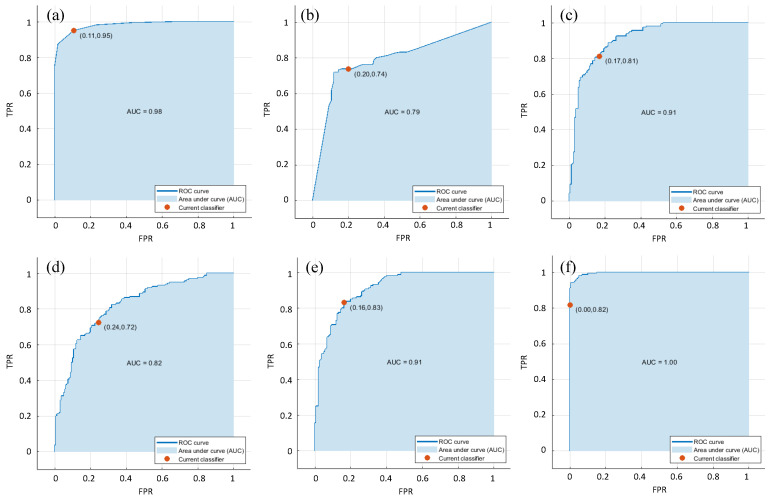
Relative operating characteristic curve (ROC) graphs and area under curves (AUCs) with different classifiers: (**a**) K nearest neighbor (KNN), (**b**) decision tree (DT), (**c**) logistic regression (LR), (**d**) linear support vector machine (SVM), (**e**) quadratic SVM and (**f**) cubic SVM.

First, the *SampEn* features across all channels are given as inputs to the different classifiers considered in this research. Figure 4 shows the highest AUC of 1 for the cubic SVM classifier with the *SampEn* features across all channels. For the same feature set, the KNN classifier also obtained a high AUC of 0.98. Additionally, it can be found that the worst AUC of 0.79 is produced in the combination DT classifier and the *SampEn* features across all channels. The 10-fold cross-validation is used in this research to assess the performance of the classifiers considered, and the performance measures are given in Table 2. As seen in Table 2, the KNN classifier achieved the highest ACC of 92.19%, highest SPE of 94.70% and highest PRE of 95.00% with the *SampEn* features across all channels. Note that the highest SEN of 100% is achieved for the cubic SVM classifier with the *SampEn* features across all channels.

**Figure 5:**
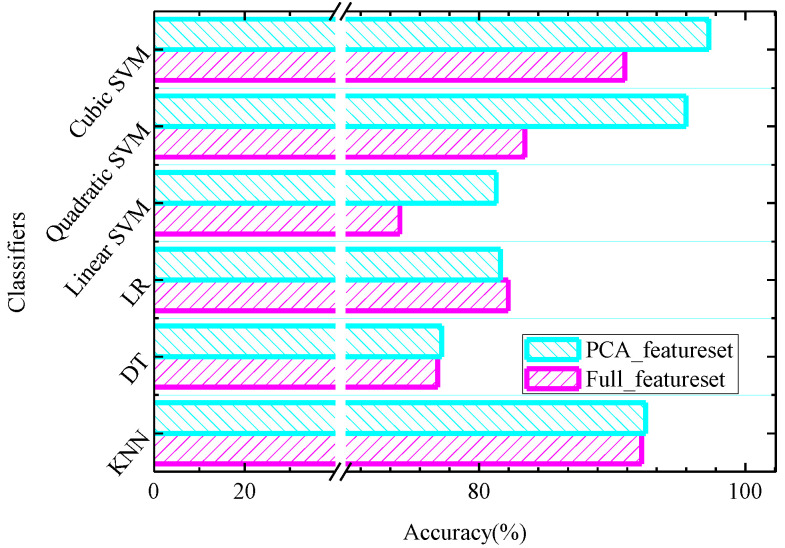
Classification accuracy of the different classifiers considered in this research based on the features before and after using the PCA method.

**Figure 6:**
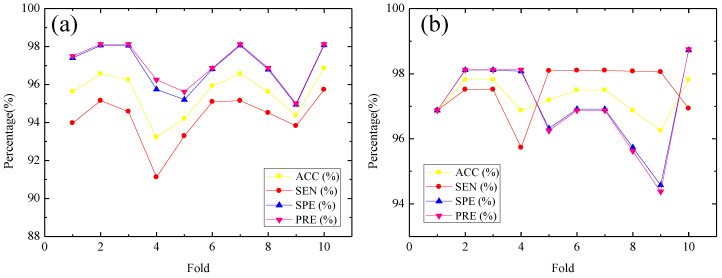
Performance plot of 10-fold cross-validation versus accuracy (ACC), sensitivity (SEN), specificity (SPE) and precision (PRE) for quadratic SVM (**a**) and cubic SVM (**b**) classifiers.

Table 3 summarizes the performance measures of the *SampEn* features across all channels after using the PCA method with the KNN, DT, LR, linear SVM, quadratic SVM and cubic SVM classifiers. Figure 5 shows classification accuracy of the different classifiers considered in this research based on the features before and after using the PCA method. For the PCA method, the component selection criterion is based on variance, and 95% variance of data is explained by the selected components. Specifically, 12 components are retained, and the variance of each component is 57.8%, 11.3%, 6.7%, 4.5%, 4.1%, 2.5%, 2.3%, 1.8%, 1.6%, 1.2%, 0.8% and 0.4% (in descending order). It can be seen from Figure 5 that improved classification accuracy has been observed for almost all classifiers considered except for the LR classifier. From Figure 5 we can also observe that with the quadratic SVM classifier, classification accuracy is increased from 83.44% to 95.52% with the PCA method, and with the cubic SVM classifier the classification accuracy is increased from 90.94% to 97.25% with the PCA method. We see that the cubic SVM classifier performs the best, followed by the quadratic SVM classifier and KNN classifier. Thus, Figure 6 summarizes detailed performance results in terms of ACC, SEN, SPE and PRE of the cubic SVM and quadratic SVM classifier for various folds in 10-fold cross-validation. From Figure 6a, it can be seen that the PCA feature set achieves a highest ACC of 96.88%, highest SEN of 95.73%, highest SPE of 98.08% and highest PRE of 98.13% for the quadratic SVM classifier at the fold of ten. In Figure 6b, the cubic SVM achieves the best performance with the highest ACC of 97.81%, SEN of 96.93%, highest SPE of 98.73% and highest PRE of 98.75% based on the PCA feature set at the fold of ten. Another test strategy is shown in Appendix A.

**Figure 7:**
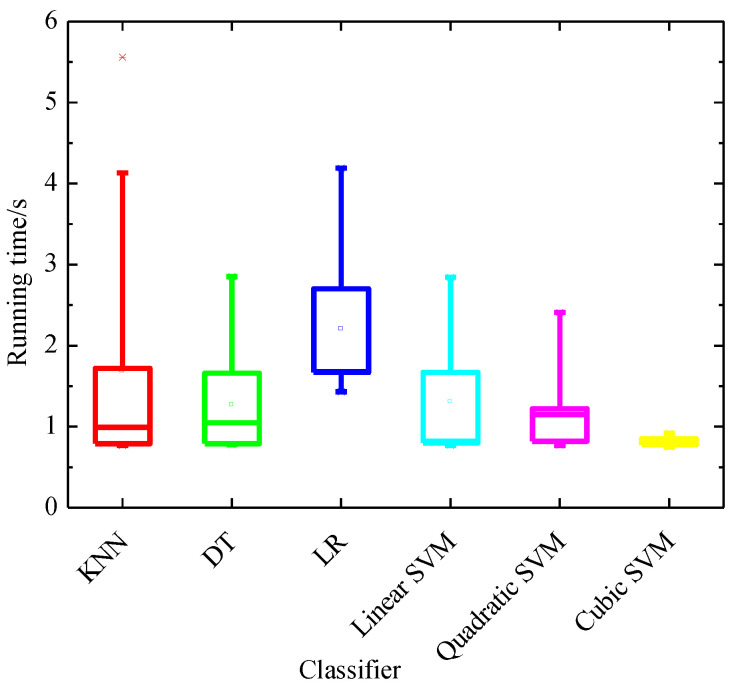
Box and whisker plots of the training time of various classifiers considered in this research upon the PCA feature.

We also evaluate the training time of various classifiers considered in this research upon the PCA feature, and the running time is computed in the environment of Matlab2018b (Massachusetts, U.S.) with Intel^®^ Core TM i5-3210 M CPU at 2.5 GHz with 6 GB RAM. In Figure 7 we present for each classifier the training time using box and whisker plots.

## 4. Discussions

For the extracted features, through comparison of running time of different classifiers, we can observe that the cubic SVM classifier is the fastest classifier in training based on the PCA feature, and the mean running time is only 0.81 s. Additionally, cubic SVM achieves the best performance with the highest ACC of 97.81%, SEN of 96.93%, highest SPE of 98.73% and highest PRE of 98.75% based on the PCA feature set at the fold of ten, indicating that PCA features combined with the cubic SVM classifier has the best performance. Additionally, the next fastest classifier in training is the quadratic SVM, which takes only 1.15 s, whereas LR is the slowest in training.

This study first proposed the *SampEn* feature across multi-channels based on EEG signals for feature extraction, then employed principal component analysis to reduce dimensionality of feature sets and, finally, adopted various classification algorithms, namely, K nearest neighbor, decision tree, support vector machine and logistic regression to identify driving-related unfavorable states. In order to demonstrate the effectiveness of our proposed method, we also employ spectral entropy and approximate entropy to extract features as a comparison. The experimental results show that the PCA spectral entropy feature combined with the cubic SVM classification algorithm obtains an accuracy of 92.50%, and the PCA-approximate entropy feature combined with the cubic SVM classification algorithm obtains an accuracy of 92.82%, which is slightly inferior to the proposed detection system considered in this research based on PCA features and the cubic SVM classification algorithm. Moreover, we compared the classification performance of our proposed methodology with some similar published works. In recent years, traffic accidents caused by unfavorable driving states have attracted the attention of a large number of scholars.

Fu et al. [2] developed a dynamic fatigue detection system by using the Hidden Markov Model (HMM) and integrated multiple physiological features to identify fatigue. Results indicated that the highest classification accuracy for this method was 95.4%. In another of their studies, Fu et al. [16] proposed a driver fatigue detection system based on electroencephalogram signals using a relative operating characteristic curve (ROC) and gray relational analysis (GRA), and the results showed that theta waves can be used as feature to identify fatigue with an AUC value of about 0.9. Wali et al. [17] classified driver distraction level by using fusion of discrete wavelet packet transform and fast Fourier transform based on wireless EEG signals, and the highest classification accuracy they achieved was 85%. In another study [26], the authors used four types of entropies measures from a single EEG channel and achieved up to an accuracy of 96.6% with a random forest classifier. In our research, we develop a detection system for unfavorable driving states based on *SampEn* features across all channels with the PCA method and achieve the highest accuracy of 97.81%, highest sensitivity of 96.93%, highest specificity of 98.73%, and highest precision of 98.75% for the cubic SVM classification algorithm. Additionally, it should also be noted that we only use a single type of entropy measures, namely *SampEn*, as a feature, and the mean training time is only 0.81 s, which is crucial for real time processing of EEG signals. The merits of our proposed detection system are as follows: (1) we achieve the highest accuracy of 97.81% for detection of unfavorable driving states in comparison with the results of some similar published works [2,16,17,26]. (2) We only use a single type of entropy measures, namely *SampEn* as a feature, and after using the PCA method our training time is much less (0.81 s). (3) Our proposed detection system based on PCA features and the cubic SVM classification algorithm shows robustness as it obtains a good sensitivity of 96.93%, specificity of 98.73% and highest precision of 98.75%.

## 5. Conclusions

Automated unfavorable states detection based on electroencephalogram (EEG) to reduce the occurrence probability of related accidents has attracted greater attention. In this research, we design a robust system to detect unfavorable driving states based on sample entropy feature analysis and multiple classification algorithms. In addition, principal component analysis (PCA) is adopted for reducing the dimensionality of feature sets. Multiple classification algorithms, namely, K nearest neighbor (KNN), decision tree (DT), support vector machine (SVM) and logistic regression (LR) are employed to improve the accuracy of unfavorable states detection. Our experimental results reveal that the feature extraction method based on sample entropy is more reliable and effective in recognizing the driver’s unfavorable state. The proposed system combines PCA and multiple classification algorithms that could improve the capacity to detect the driver’s state. In particular, the cubic SVM classification algorithm achieves the highest accuracy of 97.81%, sensitivity of 96.93%, specificity of 98.73% and precision of 98.75% in 10-fold cross-validation.

## Figures and Tables

**Table 1:** Comparative analysis of *SampEn* feature for each channel between unfavorable driving states (UDS) and non-unfavorable driving states (NUDS). The bold shows that *p* is less than 0.05.

	UDS		NUDS				UDS		NUDS		
Channels	Mean	SD	Mean	SD	*p* Value	Channels	Mean	SD	Mean	SD	*p* Value
FP1	0.488	0.141	0.495	0.134	0.814	**C4**	**0.505**	**0.128**	**0.571**	**0.182**	**0.006**
FP2	0.553	0.131	0.551	0.122	0.494	**T8**	**0.532**	**0.141**	**0.575**	**0.128**	**0.004**
**F7**	**0.436**	**0.143**	**0.483**	**0.168**	**0.021**	**TP7**	**0.447**	**0.154**	**0.527**	**0.167**	**<0.001**
**F3**	**0.447**	**0.129**	**0.511**	**0.193**	**0.004**	CP3	0.527	0.150	0.516	0.169	0.426
**FZ**	**0.411**	**0.133**	**0.482**	**0.140**	**<0.001**	**CPZ**	**0.393**	**0.154**	**0.458**	**0.163**	**<0.001**
**F4**	**0.569**	**0.157**	**0.512**	**0.136**	**0.002**	**CP4**	**0.586**	**0.156**	**0.525**	**0.145**	**0.001**
F8	0.486	0.141	0.521	0.164	0.178	TP8	0.533	0.158	0.553	0.129	0.318
**FT7**	**0.432**	**0.148**	**0.487**	**0.164**	**0.002**	P7	0.480	0.169	0.496	0.146	0.377
**FC3**	**0.474**	**0.144**	**0.526**	**0.197**	**0.027**	**P3**	**0.411**	**0.133**	**0.506**	**0.154**	**<0.001**
**FCZ**	**0.394**	**0.147**	**0.445**	**0.137**	**<0.001**	**PZ**	**0.414**	**0.163**	**0.493**	**0.159**	**<0.001**
FC4	0.519	0.161	0.509	0.153	0.545	P4	0.520	0.137	0.555	0.154	0.247
FT8	0.482	0.136	0.504	0.148	0.319	P8	0.541	0.158	0.568	0.157	0.141
**T7**	**0.462**	**0.154**	**0.522**	**0.155**	**0.001**	O1	0.459	0.146	0.465	0.122	0.231
**C3**	**0.424**	**0.127**	**0.534**	**0.159**	**<0.001**	**OZ**	**0.453**	**0.143**	**0.532**	**0.139**	**<0.001**
CZ	0.424	0.143	0.453	0.142	0.081	O2	0.485	0.146	0.505	0.152	0.081

**Table 2:** Performance measures of the *SampEn* features across all channels with the KNN, DT, LR, linear SVM, quadratic SVM and cubic SVM classifiers.

Classifiers	ACC (%)	SEN (%)	SPE (%)	PRE (%)
KNN	92.19	89.94	94.70	95.00
DT	76.88	78.67	75.29	73.75
LR	82.19	82.80	81.60	81.25
Linear SVM	74.06	74.84	73.33	72.50
Quadratic SVM	83.44	83.65	83.23	83.13
Cubic SVM	90.94	100.00	84.66	81.88

**Table 3:** Performance measures of the *SampEn* features across all channels after using the PCA method with the KNN, DT, LR, linear SVM, quadratic SVM and cubic SVM classifiers.

Classifiers	ACC (%)	SEN (%)	SPE (%)	PRE (%)
KNN	95.31	96.25	94.38	97.47
DT	89.06	90.00	88.13	88.34
LR	90.94	90.63	91.25	91.19
Linear SVM	87.81	90.63	85.00	85.80
Quadratic SVM	95.52	94.24	96.92	97.06
Cubic SVM	97.25	97.50	97.04	97.00

## Appendix A

In order to make the results more convincing, we also conducted a leave-one-subject-out cross-validation strategy. Specifically, data of 15 participants were used to train the classifiers considered in this research and then these classifiers were evaluated on the remaining one participant. This strategy was repeated 16 times to ensure that every participant was tested. Finally, we obtained an accuracy of 92.50% ± 1.73%, sensitivity of 93.00% ± 2.58%, specificity of 92.00% ± 1.63%, and precision of 92.09% ± 1.54% with the leave-one-subject-out cross-validation strategy.

## References

[1] Fu R., Wang H. (2014). Detection of driving fatigue by using noncontact emg and ecg signals measurement system. Int. J. Neural Syst..

[2] Fu R., Wang H., Zhao W. (2016). Dynamic driver fatigue detection using hidden Markov model in real driving condition. Expert Syst. Appl..

[3] Chen J., Wang H., Hua C. (2018). Electroencephalography based fatigue detection using a novel feature fusion and extreme learning machine. Cogn. Syst. Res..

[4] Kong W., Lin W., Babiloni F., Hu S., Borghini G. (2015). Investigating Driver Fatigue versus Alertness Using the Granger Causality Network. Sensors.

[5] Kar S., Bhagat M., Routray A. (2010). EEG signal analysis for the assessment and quantification of driver’s fatigue. Transp. Res. Part F Traffic Psychol. Behav..

[6] Jap B.T., Lal S., Fischer P., Bekiaris E. (2009). Using EEG spectral components to assess algorithms for detecting fatigue. Expert Syst. Appl..

[7] Correa A.G., Orosco L., Laciar E. (2014). Automatic detection of drowsiness in EEG records based on multimodal analysis. Med. Eng. Phys..

[8] Gao K., Tu H., Shi H. (2019). Stage-specific impacts of hazy weather on car following. Proc. Inst. Civ. Eng. Transp..

[9] Hao W., Daniel J. (2015). Driver Injury Severity Related to Inclement Weather at Highway-rail Grade Crossings in the United States. Traffic Inj. Prev..

[10] Chen J., Wang H., Wang Q., Hua C. (2019). Exploring the fatigue affecting electroencephalography based functional brain networks during real driving in young males. Neuropsycholoia.

[11] Shen K.-Q., Li X.-P., Ong C.-J., Shao S.-Y., Wilder-Smith E.P. (2008). EEG-based mental fatigue measurement using multi-class support vector machines with confidence estimate. Clin. Neurophysiol..

[12] Chen J., Wang H., Hua C. (2018). Assessment of driver drowsiness using electroencephalogram signals based on multiple functional brain networks. Int. J. Psychophysiol..

[13] Mu Z., Hu J., Min J. (2016). EEG-Based Person Authentication Using a Fuzzy Entropy-Related Approach with Two Electrodes. Entropy.

[14] Mu Z.D., Hu J., Yin J. (2017). Driving Fatigue Detecting Based on EEG Signals of Forehead Area. Int. J. Pattern Recognit. Artif. Intell..

[15] Chai R., Ling S.H., San P.P., Naik G.R., Nguyen T.N., Tran Y., Craig A., Nguyen H.T. (2017). Improving EEG-Based Driver Fatigue Classification Using Sparse-Deep Belief Networks. Front. Neurosci..

[16] Fu R., Wang S., Wang S. (2017). Real-time Alarm Monitoring System for Detecting Driver Fatigue in Wireless Areas. Promet Traffic Transp..

[17] Wali M.K., Murugappan M., Ahmmad B. (2013). Wavelet Packet Transform Based Driver Distraction Level Classification Using EEG. Math. Probl. Eng..

[18] Simon M., Schmidt E.A., Kincses W.E., Fritzsche M., Bruns A., Aufmuth C., Bogdan M., Rosenstiel W., Schrauf M. (2011). EEG alpha spindle measures as indicators of driver fatigue under real traffic conditions. Clin. Neurophysiol..

[19] Da Silveira T.L.T., Kozakevicius A.J., Rodrigues C.R. (2016). Automated drowsiness detection through wavelet packet analysis of a single EEG channel. Expert Syst. Appl..

[20] Arunkumar N., Ramkumar K., Venkatraman V., Abdulhay E., Fernandes S.L., Kadry S., Segal S. (2017). Classification of focal and non focal EEG using entropies. Pattern Recognit. Lett..

[21] Liu M., Liu X., Hildebrandt A., Zhou C. (2020). Individual Cortical Entropy Profile: Test–Retest Reliability, Predictive Power for Cognitive Ability, and Neuroanatomical Foundation. Cereb. Cortex Commun..

[22] Carhart-Harris R.L., Leech R., Hellyer P.J., Shanahan M., Efeilding A., Etagliazucchi E., Chialvo D.R., Enutt D. (2014). The entropic brain: A theory of conscious states informed by neuroimaging research with psychedelic drugs. Front. Hum. Neurosci..

[23] Keshmiri S. (2020). Entropy and the Brain: An Overview. Entropy.

[24] Mu Z., Hu J., Min J. (2017). Driver Fatigue Detection System Using Electroencephalography Signals Based on Combined Entropy Features. Appl. Sci..

[25] Huang H.B., Li R.X., Huang X.R., Lim T.C., Ding W.P. (2016). Identification of vehicle suspension shock absorber squeak and rattle noise based on wavelet packet transforms and a genetic algorithm-support vector machine. Appl. Acoust..

[26] Hu J. (2017). Comparison of Different Features and Classifiers for Driver Fatigue Detection Based on a Single EEG Channel. Comput. Math. Methods Med..

[27] Chattopadhyay S., Banerjee S., Rabhi F.A., Acharya U.R. (2012). A Case-Based Reasoning system for complex medical diagnosis. Expert Syst..

[28] Birant D. (2011). Comparison of Decision Tree Algorithms for Predicting Potential Air Pollutant Emissions with Data Mining Models. J. Environ. Inform..

[29] Yeo M.V., Li X., Shen K., Wilder-Smith E.P. (2009). Can SVM be used for automatic EEG detection of drowsiness during car driving?. Saf. Sci..

[30] Michalaki P., Quddus M.A., Pitfield D., Huetson A. (2015). Exploring the factors affecting motorway accident severity in England using the generalised ordered logistic regression model. J. Saf. Res..

